# TRPV1 and TRPA1 in Lung Inflammation and Airway Hyperresponsiveness Induced by Fine Particulate Matter (PM_2.5_)

**DOI:** 10.1155/2019/7450151

**Published:** 2019-06-02

**Authors:** Mengmeng Xu, Yanbei Zhang, Muyun Wang, Hai Zhang, Yuqing Chen, Ian M. Adcock, Kian Fan Chung, Jinhan Mo, Yinping Zhang, Feng Li

**Affiliations:** ^1^Department of Pulmonary Medicine, Shanghai Chest Hospital, Shanghai Jiao Tong University, Shanghai 200030, China; ^2^Department of Respiratory and Critical Care Medicine, The Geriatric Institute of Anhui, The First Affiliated Hospital of Anhui Medical University, Hefei, Anhui 230032, China; ^3^Airway Disease Section, National Heart and Lung Institute, Imperial College London, London SW3 6LY, UK; ^4^Department of Building Science, Tsinghua University, Beijing 100084, China

## Abstract

Exposure to fine particulate matter (PM_2.5_) has been associated with lung inflammation and airway hyperresponsiveness (AHR). Transient receptor potential (TRP) vanilloid 1 (TRPV1) and ankyrin 1 (TRPA1) both may play important roles in lung inflammation and AHR. We investigated whether PM_2.5_-induced lung inflammation and AHR could be prevented by blocking TRPV1 and TRPA1 channels. Mice were injected intraperitoneally with AMG9810 (30 mg/kg, a TRPV1 antagonist) or A967079 (30 mg/kg, a TRPA1 antagonist) or their combination or vehicle (PBS) one hour before intranasal instillation of PM_2.5_ (7.8 mg/kg) or vehicle (PBS) for two consecutive days, and then the mice were studied 24 h later. All pretreatments inhibited PM_2.5_-induced AHR and inflammatory infiltration in the lung tissue and decreased inflammatory cytokine levels in the bronchoalveolar lavage fluid, together with oxidant levels in the lung. AMG9810 inhibited MFF expression and increased MFN2 expression while A967079 inhibited DRP1 expression and increased OPA1 expression; combined pretreatment reduced MFF and DPR1 expression and increased MFN2 and OPA1 expression. All pretreatments inhibited the activation of the TLR4/NF-*κ*B pathway, while A967079 alone, and combined with AMG9810 also reduced the activation of the NLRP3/caspase-1 pathway. Both TRPV1 and TRPA1 channels play an important role in PM_2.5_-induced lung inflammation and AHR. However, inhibition of the TRPA1 channel or combined inhibition of TRPA1 and TRPV1 channels resulted in greater inhibitory effect on PM_2.5_-induced lung injury through regulating the mitochondrial fission/fusion proteins and inhibiting the TLR4/NF-*κ*B and NLRP3/caspase-1 pathways.

## 1. Introduction

Due to increasing urbanization and modernization, particulate matter (PM) pollution has become a serious health risk in China. Fine particulate matter (PM_2.5_, particle size < 2.5*μ*m) generally has greater toxicity than other particles due to its smaller diameter and larger surface-to-mass ratio, thus allowing it to enter into the lower respiratory tract and even penetrate into the alveolar space and ultimately into the blood circulation [1]. PM_2.5_ exposure can induce lung inflammation and airway hyperresponsiveness (AHR) and can even increase the risk of developing asthma [2, 3]. The underlying mechanisms of PM_2.5_ toxicity may be partly explained by its ability to cause oxidative stress and mitochondrial damage [4], a process that involves the activation of pattern recognition receptors (PRRs), such as Toll-like receptors (TLRs) and nucleotide binding domain leucine-rich repeat-containing receptors (NLRs) [5]. Activated PPRs may initiate a series of intracellular signaling events which include the activation of TLR4/NF-*κ*B and NLRP3/caspase-1 pathways, as demonstrated in our previous study [6].

Mitochondria are double-membraned and multifunctional cellular organelles that play an important role in cell morphology and physiology including synthesis of adenosine triphosphate (ATP), redox homeostasis, cellular metabolism, and apoptosis [7]. The morphology of mitochondria is regulated by fission and fusion processes. The former is mainly coordinated by dynamin-related protein-1 (DRP1) within the cytoplasm, as well as mitochondria fission factor (MFF) and fission 1 (FIS1) on the outer mitochondrial membrane to facilitate mitochondrial mobility and eliminate dysfunctional mitochondria. The latter is controlled by mitofusin (MFN1 and MFN2) localized on the outer mitochondrial membrane and optic atrophy 1 (OPA1) from the inner mitochondrial membrane to produce an elaborately interconnected reticulum and promote cristae formation [8]. Mitochondria can generate reactive oxygen species (ROS), i.e., mitochondrial ROS (mtROS). Mitochondria are also highly sensitive to oxidative stress as this may in turn induce mitochondrial damage [6, 9, 10].

Transient receptor potential (TRP) vanilloid 1 (TRPV1) and ankyrin 1 (TRPA1), members of the TRP channel superfamily, are coexpressed in nociceptive C fibers innervating the airways and are also found in airway epithelial cells (AECs) and airway smooth muscle(ASM) cells [11]. The TRPV1 channel can be activated by capsaicin, acidic pH, and oxidative stress [12], while the TRPA1 channel can be activated by ROS such as superoxide, hydrogen peroxide (H_2_O_2_), and the products of lipid peroxidation [13]. Moreover, both channels can be activated by exogenous environmental irritants, such as diesel exhaust particles (DEP), ozone, cigarette smoke, and PMs [11]. The activation of TRPV1 and TRPA1 could induce airway neurogenic inflammation with the release of inflammatory neuropeptides including neurokinin A (NKA), substance P, and calcitonin gene-related peptide (CGRP), as well as inflammatory mediators such as ATP, leukotrienes, TNF-*α*, and IL-1*β*, which may drive the early airway inflammation and ASM contraction [14, 15]. Previous studies indicated that activation of TRPV1 and TRPA1 stimulated airway nociceptive C fibers and caused cough in guinea pigs and humans [15, 16].

In the present study, we examined the importance and mechanism of TRPV1 and TRPA1 actions in the PM_2.5_-induced murine model of lung inflammation, AHR, and oxidative stress by administering AMG9810, a TRPV1 antagonist, and A967079, a TRPA1 antagonist, separately or in combination prior to PM_2.5_ instillation to mice.

## 2. Materials and Methods

### 2.1. PM_2.5_ Sampling, Extraction, and Chemical Analysis

PM_2.5_ samples were collected on quartz filters (Tissuquartz, Pall, USA) using a high flow volume PM_2.5_ Sampler (Ecotech, Australia) at a flow rate of 1.13 m^3^/min, located on the top of a building in Xuhui District in Shanghai, China, from September 2017 to April 2018. The filters were cut into small fragments (1 cm × 3 cm), then immersed into ultrapure deionized water and eluted with an ultrasonic cleaner (KUDOS, Shanghai, China), followed by freeze-drying with a vacuum freeze dryer ( Four-Ring Science Instrument Plant, Beijing, China). Finally, PM_2.5_ solid particulates were collected and preserved at -80°C until required. The particulates were quantified and suspended evenly in phosphate-buffered saline (PBS) by vortex oscillation before intranasal instillation.

A portion of particulates was extracted with 18 MΩ Milli-Q water in a sonication ice-water bath. After being filtered, the water extracts were subjected to analysis of inorganic anions (e.g., F^−^, NO_3_^−^, Cl^−^, and SO_4_^2−^) and cations (e.g., Na^+^, NH_4_^+^, K^+^, Ca^2+^, and Mg^2+^) using an ion chromatography system. Parts of extracted PM_2.5_ solid particulates were taken for the measurements of TOC using a total organic carbon (TOC) analyzer (multi N/C2100, Analytik Jena, Germany). For the analysis of polycyclic aromatic hydrocarbons (PAHs) in PM_2.5_, the particulates were sonicated in dichloromethane (DCM)/methanol (1 : 1, *v*/*v*), concentrated to approximately 1 mL by rotary evaporator and then blown to 200 *μ*L under a gentle stream of nitrogen. Finally, the methylated particles were analyzed with a gas chromatography-mass spectrometer (Agilent, Alpharetta, GA, USA). Endotoxin content in PM_2.5_ was quantified using a quantitative kinetic chromogenic LAL test kit (Lonza, Switzerland) according to the instructions from the manufacturer.

### 2.2. PM_2.5_ Instillation and Inhibitor Administration

Sixty-four 8-week-old male C57/BL6 mice, weight 22-25 g, were purchased from Shanghai Super–B&K Laboratory Animal Corporation (Shanghai, China). All the mice were housed in a specific pathogen-free facility where the circulating temperature is 22°C with 50-60% humidity, equal light-dark cycle, and with access to standard food and water ad libitum. All experimental studies involving animals were approved by the laboratory animal ethics committee of the institute.

Mice were administered intraperitoneally with AMG9810 (30 mg/kg, dissolved in saline including 2% DMSO and 5% Tween-80, Abcam, Cambridge, MA, USA) [17] or A967079 (30 mg/kg, dissolved in saline including 30% DMSO and 30% PEG400, Abcam, USA) [18] or their combination one hour before intranasal instillation of 50 *μ*L of PM_2.5_ suspension (7.8 mg/kg) [6] or vehicle (PBS) once a day for two consecutive days. There were eight groups with eight mice within each group: group 1, PBS+PBS; group 2, AMG9810+PBS; group 3, A967079+PBS; group 4, AMG9810+A967079+PBS; group 5, PBS+PM_2.5_; group 6, AMG9810+PM_2.5_; group 7, A967079+PM_2.5_; and group 8, AMG9810+A967079+PM_2.5_.

### 2.3. AHR

After anesthesia with an intraperitoneal injection of 0.2 mL 1% pentobarbital, mice were tracheostomized and placed in a whole-body plethysmograph with aerosol inhalation of acetylcholine (ACh) for the measurement of airway resistance and compliance (EMMS, Hants, UK). The concentration of ACh required to increase lung resistance by 200% from baseline was calculated as PC200, and -logPC_200_ was taken as a measure of airway responsiveness.

### 2.4. Bronchoalveolar Lavage (BAL) Fluid Collection, Cell Counting, and Cytokine Assay

Following terminal aesthesia with pentobarbitone, mice were lavaged with 2 mL of PBS via an endotracheal tube. The bronchoalveolar lavage (BAL) fluid was centrifuged at 4°C and 1000 rpm for 10 min. The supernatant was stored and the cell pellet was resuspended in PBS. Total cell counts were determined using a hemocytometer, and differential cell counts from cytospin preparations stained by Liu's stain solution (BaSO Diagnostics Inc., Zhuhai, China) were measured under a microscope. At least 500 cells were counted and identified as macrophages, lymphocytes, neutrophils, or eosinophils according to standard morphology.

The levels of TNF-*α*, chemokine (C-X-C motif) ligand 1 (KC), IL-1*β*, and IL-6 in BAL fluid were measured with corresponding ELISA kits (Mutisciences, Hangzhou, China) following the instructions from the manufacturer.

### 2.5. Histological Analysis and Immunohistochemistry

The whole lung was removed, and the right lung lobe was dissected and snap-frozen in liquid nitrogen for later analysis. The left lung was inflated with 4% paraformaldehyde under 25 cm of water pressure and then embedded in paraffin. Paraffin blocks were sectioned to expose the maximum surface area of the lung tissue in the plane of the bronchial tree. Four *μ*m sections were cut and stained with hematoxylin and eosin (H&E).

The extent of lung inflammation was evaluated in the H&E-stained lung sections as described previously [19] using the following scale: 0 = no inflammatory response, 1 = mild inflammation with foci of inflammatory cells in the bronchial or vascular wall and in alveolar septa, 2 = moderate inflammation with patchy inflammation or localized inflammation in walls of the bronchi or blood vessels and alveolar septa and less than 1/3 of the lung cross-sectional area is involved, and 3 = severe inflammation with diffuse inflammatory cells in walls of the bronchi or blood vessels and alveoli septa; between one-third and two-thirds of the lung area are involved.

The localization and expression of TRPV1 and TRPA1 were examined by immunohistochemical staining. Lung sections were incubated with anti-TRPV1 or anti-TRPA1 primary antibody (Novus Biologicals, Littleton, Colorado, USA) and polyclonal goat anti-rabbit horseradish peroxidase-conjugated secondary antibody followed by diaminobenzidine liquid. The immunostaining intensity for TRPV1 and TRPA1 in lung tissues was scored on a 0-3 scale [6].

### 2.6. Oxidant Levels in the Lung Tissue

Fresh lung tissue homogenates were extracted, and then protein concentrations were measured using a BCA assay kit (Thermo Fisher Scientific, Waltham, MS, USA). The levels of malondialdehyde (MDA) and hydrogen peroxide (H_2_O_2_) in lung tissues were respectively measured with corresponding assay kits (Nanjing Jiancheng Bioengineering Institute, Nanjing, Jiangsu, China) according to the manufacturer's instructions.

The mitochondria of fresh lung tissues were extracted with a Tissue Mitochondria Isolation Kit (Beyotime Biotechnology, Haimen, Jiangsu, China), then resuspended with mitochondrial stock solutions and qualified using a BCA assay kit (Thermo Scientific, USA). Immediately, equal amounts of mitochondrial suspension were incubated with 5 *μ*M MitoSOX working solution for 10 min at 37°C whilst being protected from light. MitoSOX fluorescence was measured by Varioskan Flash (Thermo Scientific, USA) at wavelengths of 510 nm for excitation and 580 nm for emission.

### 2.7. Western Blot Analysis

Total lung tissue proteins were homogenized with a RIPA lysis buffer (Beyotime Biotechnology, China), and protein concentrations were quantified by a BCA assay kit (Thermo Fisher Scientific, USA). 30 *μ*g of proteins per lane was separated through 10–15% denaturing polyacrylamide gels and transferred to PVDF membranes. The membranes were blocked with 5% nonfat dry milk and incubated with the following primary antibodies: TRAV1 and TRPA1 (Novus Biologicals, USA), MFF, DRP1, MFN2, OPA1, phosphorylated (phospho) NF-*κ*B P65, total NF-*κ*B P65, NLRP3 (Cell Signaling Technology, Danvers, MA, USA), and caspase-1 (Abcam, USA) overnight at 4°C. Membranes were then incubated with an HRP-conjugated anti-rabbit secondary antibody (Cell Signaling Technology, USA) and then visualized by chemiluminescent detection.

### 2.8. Caspase-1 Activity in the Lung Tissue

Caspase-1 activity in the lung tissue was detected using a Caspase-1 Activity Assay Kit (Beyotime Biotechnology, China). Lung tissues were homogenized and then centrifuged at 15,000 rpm/min for 15 min at 4°C. The supernatants were collected and qualified by a BCA assay kit, and caspase-1 activity in an equal amount of protein of about 200 *μ*g was determined immediately. In brief, the substrate, Ac-YVAD-pNA, was added to the supernatant and incubated for 60-120 min at 37°C. When the solution showed an obvious yellow pNA colour, the reaction was stopped and the sample assayed by Varioskan Flash (Thermo Fisher Scientific, USA) at 405 nm. The level of caspase-1 activity was quantified using a standard curve.

### 2.9. Statistical Analysis

All results were expressed as a mean ± S.E.M. To compare the differences between three interventions on control mice and to compare the differences between control mice and model mice, group 1 to group 5 were compared together. To compare the differences between three interventions on model mice and to compare the differences between control mice and model mice, group 5 to group 8 were compared together. Two-way ANOVA was performed for comparisons of % change in lung resistance between individual groups. One-way ANOVA with Bonferroni's post hoc test (for equal variance) or Dunnett's T3 post hoc test (for unequal variance) was performed for comparisons among multiple groups. *P* < 0.05 was considered significant.

## 3. Results

### 3.1. Chemical Analysis of PM_2.5_

The analyzed results demonstrated there were metal ions, oxidizing ions, PAHs and endotoxin in PM_2.5_ samples (Table 1).

### 3.2. AHR Measurements

**Figure 1:**
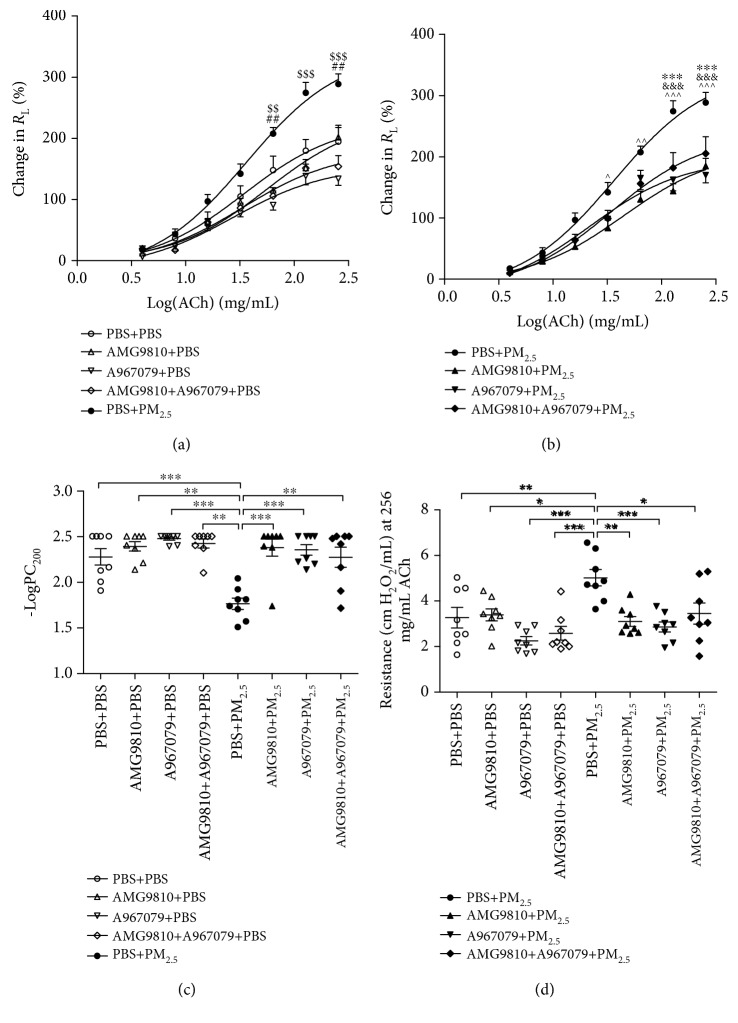
Mean percentage increase in lung resistance (*R*_L_) to increasing concentrations of acetylcholine (ACh) (a, b). ^##^*P* < 0.01 compared with A967079+PBS mice and ^$$^*P* < 0.01 and ^$$$^*P* < 0.001 compared with PBS+PM_2.5_ mice (a). ^∧^*P* < 0.05, ^∧∧^*P* < 0.01, and ^∧∧∧^*P* < 0.001 compared with AMG9810+PM_2.5_ mice, ^&&&^*P* < 0.001 compared with A967079+PM_2.5_ mice, and ^∗∗∗^*P* < 0.001 compared with AMG9810+A967079+PM_2.5_ mice (b). -logPC_200_ was measured as an indicator of bronchial responsiveness. Individual and mean -logPC_200_ (c). Individual and mean airway resistance at 256 mg/L of acetylcholine (ACh) (d). ^∗^*P* < 0.05, ^∗∗^*P* < 0.01, and ^∗∗∗^*P* < 0.001 compared with PBS+PM_2.5_ mice.

PBS-pretreated PM_2.5_-instilled mice demonstrated a significant leftward shift of the concentration-response curve (Figure 1(a)) with a decreased value of -logPC_200_, indicating an increase in airway responsiveness to the ACh challenge (Figure 1(c)) and increased lung resistance (Figure 1(d)) compared to PBS-treated PBS-instilled mice. In PBS-instilled mice, pretreatment with AMG9810, A967079, or their combination had no effect on airway responsiveness in terms of -logPC_200_ and lung resistance at 256 mg/L of ACh compared to that in PBS-treated PBS-instilled mice (Figures 1(c) and 1(d)), although there were decreases in lung resistance at 64 mg/L and 256 mg/L of ACh in the A967079-pretreated PBS-instilled group compared with the PBS-pretreated and PBS-instilled group (Figure 1(a)). In PM_2.5_-instilled mice, pretreatment with AMG9810, A967079, or their combination reduced airway responsiveness in terms of -logPC_200_ and airway resistance compared to that in PBS-pretreated PM_2.5_-instilled mice (Figures 1(b)–1(d)).

### 3.3. BAL Cells

**Figure 2:**
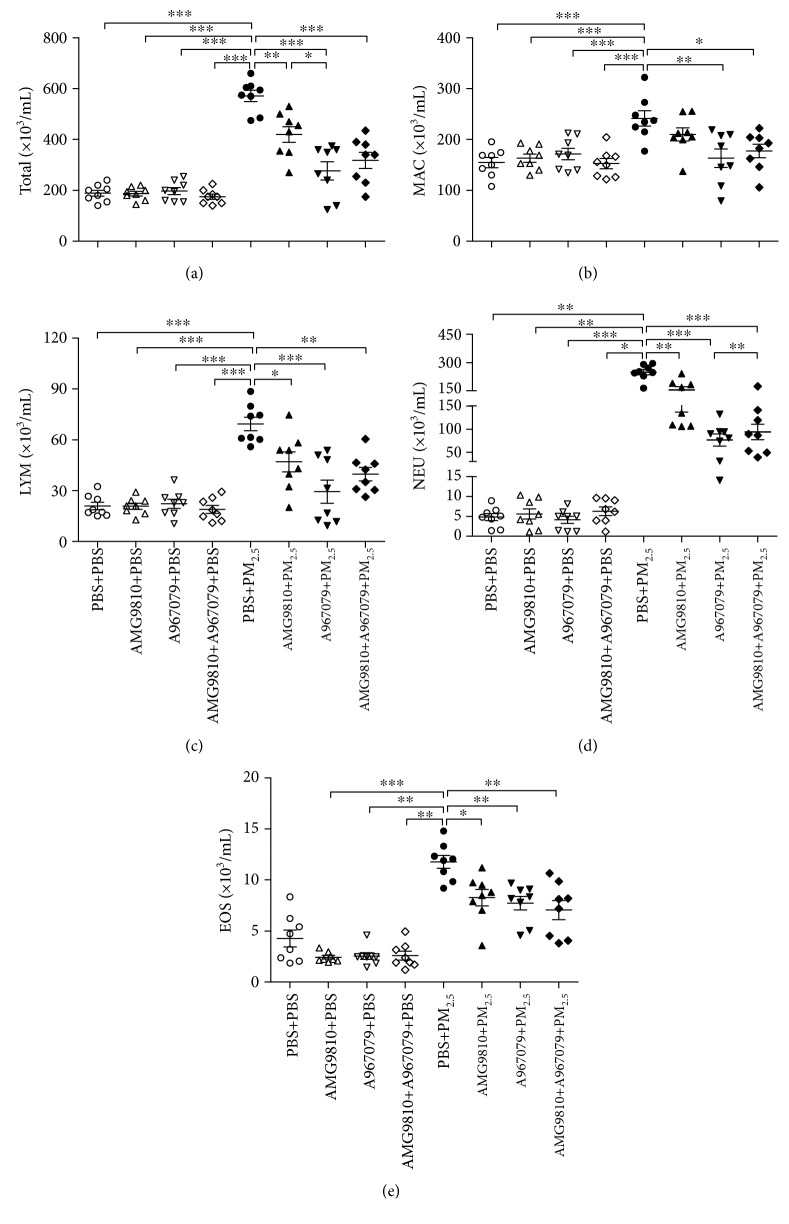
Individual and mean numbers of total cells (Total) (a), macrophages (MAC) (b), lymphocytes (LYM) (c), neutrophils (NEU) (d), and eosinophils (EOS) (e) in BAL fluid. ^∗^*P* < 0.05, ^∗∗^*P* < 0.01, and ^∗∗∗^*P* < 0.001 compared with PBS+PM_2.5_ mice.

There were increases in total cells including macrophages, lymphocytes, and neutrophils in the BAL fluid of PBS-pretreated PM_2.5_-instilled mice compared with that of PBS-pretreated PBS-instilled mice (Figures 2(a)–2(d)). In PBS-instilled mice, pretreatment with AMG9810 or A967079 or their combination did not affect inflammatory cell numbers compared with that in PBS-pretreated PBS-instilled mice (Figures 2(a)–2(e)). In PM_2.5_-instilled mice, pretreatment with AMG9810 reduced the number of total cells, lymphocytes, neutrophils, and eosinophils (Figures 2(a) and 2(c)–2(e)) and pretreatment with A967079 or combined with AMG9810 further reduced the number of macrophages (Figures 2(a)–2(e)). In addition, pretreatment with A967079 reduced more total cells and neutrophils than pretreatment with AMG9810 in PM_2.5_-instilled mice (Figures 2(a) and 2(d)).

### 3.4. Lung Histological Changes

**Figure 3:**
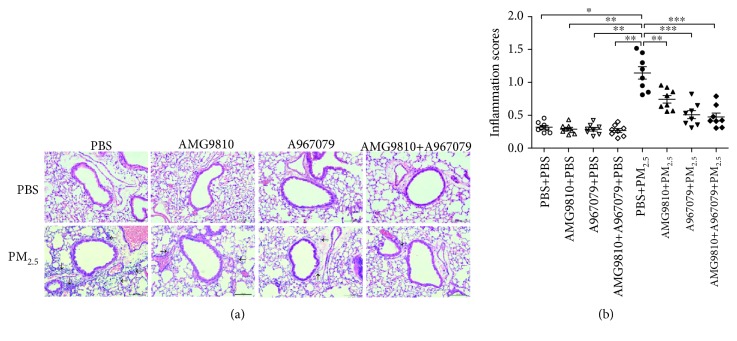
Representative bronchial photomicrographs of mouse lung tissues in hematoxylin and eosin- (H&E-) stained sections from PBS-pretreated, AMG9810-pretreated, A967079-pretreated, and AMG9810+A967079-pretreated PBS-instilled mice and PBS-pretreated, AMG9810-pretreated, A967079-pretreated, and AMG9810+A967079-pretreated PM_2.5_-instilled mice (bar = 100*μ*m) (a). Individual and mean values of inflammation scores measured from H&E-stained sections (b). ^∗^*P* < 0.05, ^∗∗^*P* < 0.01, and ^∗∗∗^*P* < 0.001 compared with PBS+PM_2.5_ mice. Black arrows show inflammatory cells along the bronchus and alveolar septa.

Examples of the lung tissue with infiltration of inflammatory cells around the bronchus and vessel after instillation of PM_2.5_ are shown in Figure 3(a). There were higher inflammation scores in PBS-pretreated PM_2.5_-instilled mice compared with PBS-pretreated PBS-instilled mice (Figure 3(b)). Pretreatment with AMG9810, A967079, or their combination to PBS-instilled mice did not change lung inflammation scores compared with that to PBS-pretreated PBS-instilled mice (Figure 3(b)). Pretreatment with AMG9810, A967079, or their combination reduced inflammation scores in PM_2.5_-instilled mice compared with PBS-pretreated PM_2.5_-instilled mice (Figure 3(b)).

### 3.5. Cytokine Levels in BAL Fluid

**Figure 4:**
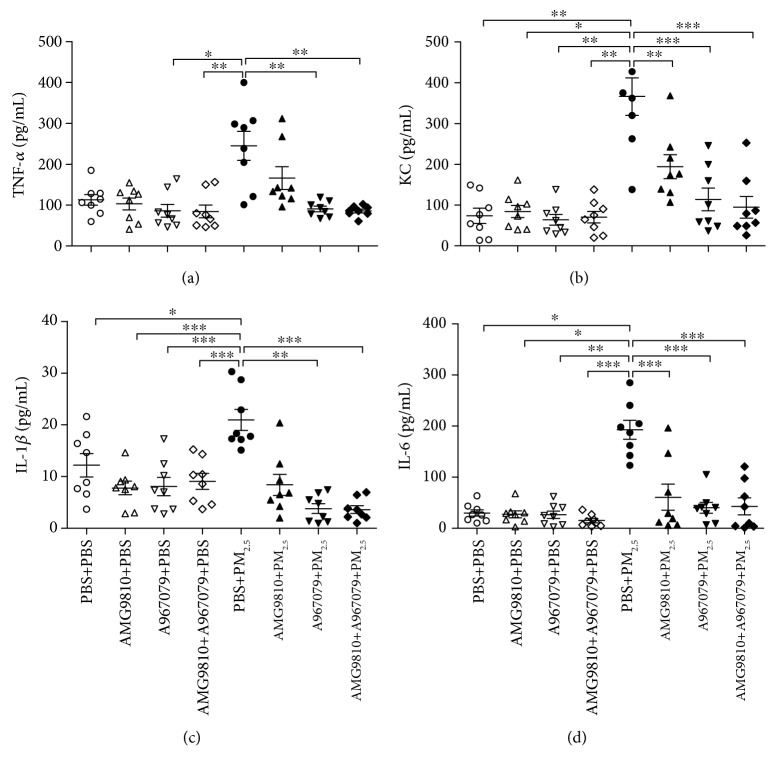
Individual and mean levels of TNF-*α* (a), chemokine (C-X-C motif) ligand 1 (KC) (b), IL-*β* (c), and IL-6 (d) in bronchoalveolar lavage fluid (BALF). ^∗^*P* < 0.05, ^∗∗^*P* < 0.01, and ^∗∗∗^*P* < 0.001 compared with PBS+PM_2.5_ mice.

PBS-pretreated PM_2.5_-instilled mice demonstrated increased levels of KC, IL-1*β*, and IL-6 in BAL fluid compared with PBS-pretreated PBS-instilled mice (Figures 4(b)–4(d)). In PBS-instilled mice, there were no significant changes in the levels of TNF-*α*, KC, IL-1*β*, and IL-6 by pretreatment with AMG9810, A967079, or their combination compared with those of PBS-pretreated PBS-instilled mice (Figures 4(a)–4(d)). In PM_2.5_-instilled mice, AMG9810 pretreatment reduced the levels of KC and IL-6 in the BAL fluid and pretreatment with A967079 or combined with AMG9810 reduced the levels of TNF-*α*, KC, IL-1*β*, and IL-6 compared with that in PBS-pretreated PM_2.5_-instilled mice (Figures 4(a)–4(d)).

### 3.6. MDA, H_2_O_2_, and mtROS Levels

**Figure 5:**
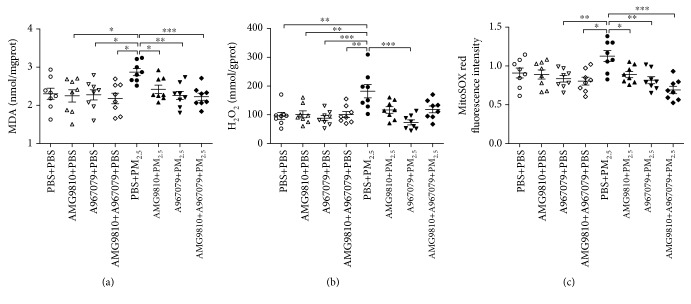
Individual and mean levels of malondialdehyde (MDA) (a) and hydrogen peroxide (H_2_O_2_) (b) in lung tissue homogenates. Individual and relative levels of mitochondrial ROS (mtROS) in lung mitochondrial suspensions (c). ^∗^*P* < 0.05, ^∗∗^*P* < 0.01, and ^∗∗∗^*P* < 0.001 compared with PBS+PM_2.5_ mice.

In PBS-pretreated PM_2.5_-instilled mice, there was increased lung MDA compared with that in AMG9810-, A967079-, or combined AMG9810 and A967079-pretreated PBS-instilled mice; increased lung H_2_O_2_ compared with that in PBS-, AMG9810-, A967079-, or combined AMG9810 and A967079-pretreated PBS-instilled mice; and increased lung mtROS compared with that in A967079- or combined AMG9810 and A967079-pretreated PBS-instilled mice (Figures 5(a)–5(c)). Pretreatment with AMG9810, A967079, or their combination in PBS-instilled mice showed no effects on levels of MDA, H_2_O_2_, and mtROS compared with that in PBS-pretreated PBS-instilled mice (Figures 5(a)–5(c)). Pretreatment with AMG9810 or combined with A967079 reduced MDA and mtROS levels, and pretreatment with A967079 reduced MDA, H_2_O_2_, and mtROS in PM_2.5_-instilled mice compared with PBS-treated PM_2.5_-instilled mice (Figures 5(a)–5(c)).

### 3.7. TRPV1 and TRPA1 Expression in the Lung Tissue

**Figure 6:**
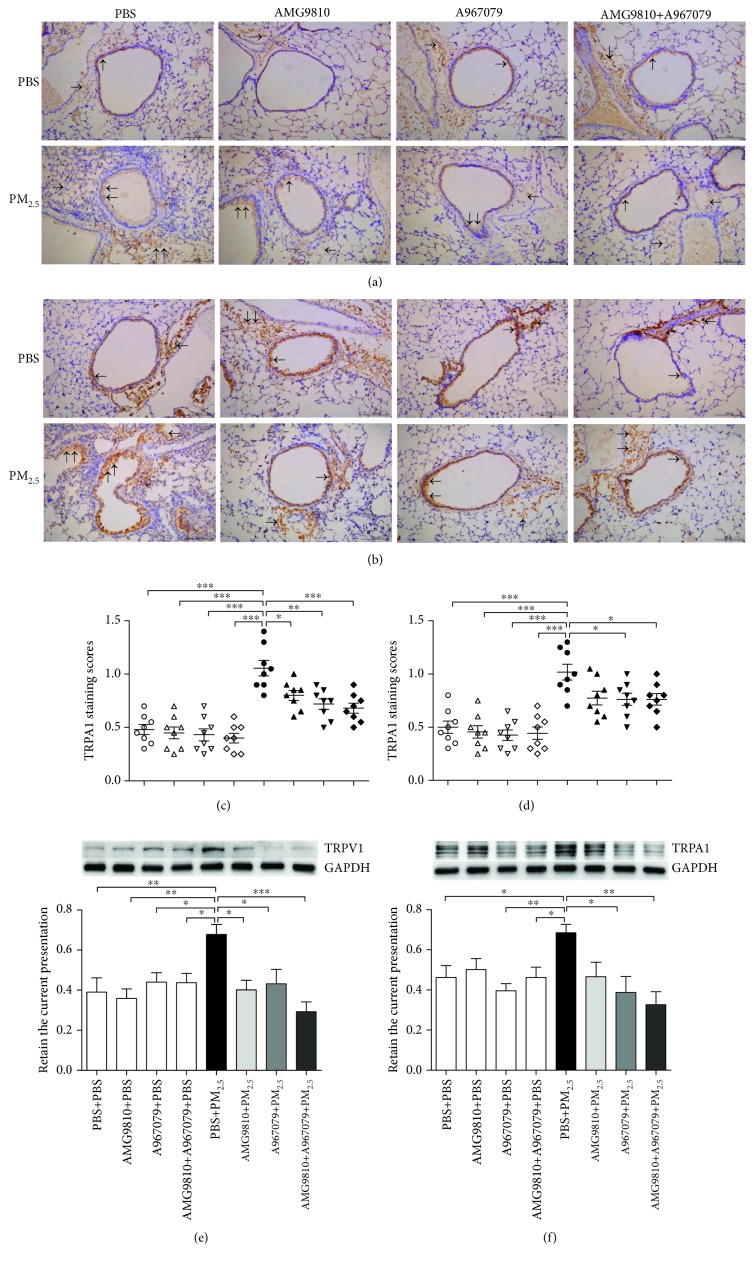
Representative immunohistochemical (IHC) staining for TRPV1 (a) and TRPA1 (b) as indicated by the brown stains (arrows) in mouse lung tissue sections from PBS-pretreated, AMG9810-pretreated, A967079-pretreated, and AMG9810+A967079-pretreated PBS-instilled mice and from PBS-pretreated, AMG9810-pretreated, A967079-pretreated, and AMG9810+A967079-pretreated PM_2.5_-instilled mice (bar = 100*μ*m). Individual and mean immunostaining scores of TRPV1 (c) and TRPA1 (d) measured from lung immunohistochemical sections. Western blot analysis of the relative protein expression of TRPV1 (e) and TRPA1 (f) to GAPDH in mouse lung tissue homogenates (f). ^∗^*P* < 0.05, ^∗∗^*P* < 0.01, and ^∗∗∗^*P* < 0.001 compared with PBS+PM_2.5_ mice.

As indicated by immunohistochemical staining (Figures 6(a) and 6(b)), the expressions of TRPV1 and TRPA1 were mainly distributed in the airway epithelium and ASM layer. PBS-pretreated PM_2.5_-instilled mice demonstrated notable increases in TRPV1 and TRPA1 immunostaining scores compared to PBS-pretreated PBS-instilled mice (Figures 6(c) and 6(d)), which were consistent with the changes on Western blot analysis (Figures 6(e) and 6(f)). In PBS-instilled mice, pretreatment with AMG9810, A967079, or their combination showed no effects on the TRPV1 and TRPA1 expression compared with that in PBS-pretreated PBS-instilled mice. In PM_2.5_-instilled mice, pretreatment with A967079 or combined A967079 and AMG9810 reduced the expression of TRPV1 and TRPA1; pretreatment with AMG9810 reduced only the TRPV1 expression (Figures 6(c)–6(f)).

### 3.8. Mitochondrial Fission/Fusion Protein Expression in the Lung Tissue

**Figure 7:**
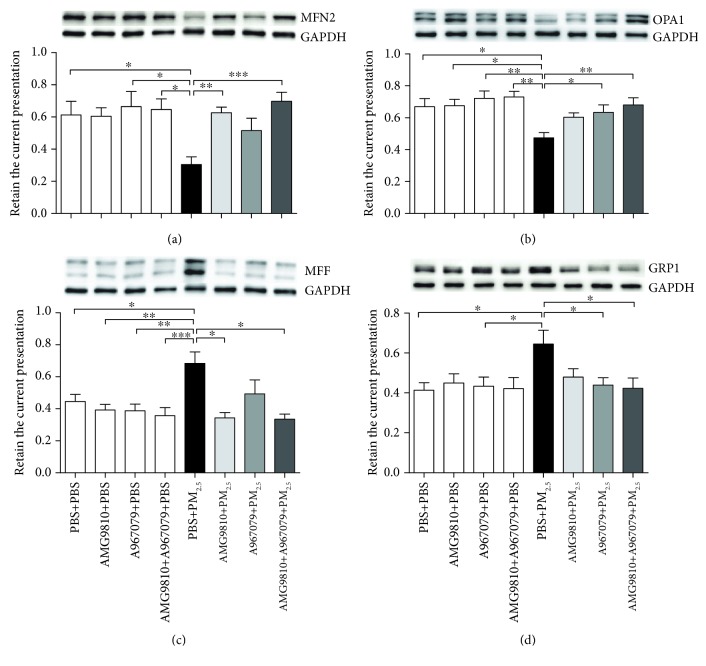
Western blot analysis of the relative protein expression of the mitochondrial fission factor (MFF) (a), dynamin-related protein 1 (DRP1) (b), mitofusin 2 (MFN2) (c), and optic atrophy 1 (OPA1) (d) to glyceraldehyde-3-phosphate dehydrogenase (GAPDH) in mouse lung tissue homogenates. Each panel shows a representative Western blot. ^∗^*P* < 0.05, ^∗∗^*P* < 0.01, and ^∗∗∗^*P* < 0.001 compared with PBS+PM_2.5_ mice.

PBS-pretreated PM_2.5_-instilled mice demonstrated increased protein expression of MFF and DRP1 and decreased protein expression of MFN2 and OPA1 compared with PBS-pretreated PBS-instilled mice (Figures 7(a)–7(d)). The expression of mitochondrial fission/fusion protein was not affected by pretreatment with AMG9810, A967079, or their combination in PBS-instilled mice. In PM_2.5_-instilled mice, pretreatment with AMG9810, A967079, or their combination resulted in a trend towards a return to control levels in terms of mitochondrial fission and fusion proteins. AMG9810 inhibited MFF expression and increased MFN2 expression, A967079 inhibited DRP1 expression and increased OPA1 expression, and the combined pretreatment downregulated the MFF and the DRP1 expression and enhanced the MFN2 and the OPA1 expression (Figures 7(a)–7(d)).

### 3.9. Activation of TLR4/NF-*κ*B and NLRP3/Caspase-1 Pathways

**Figure 8:**
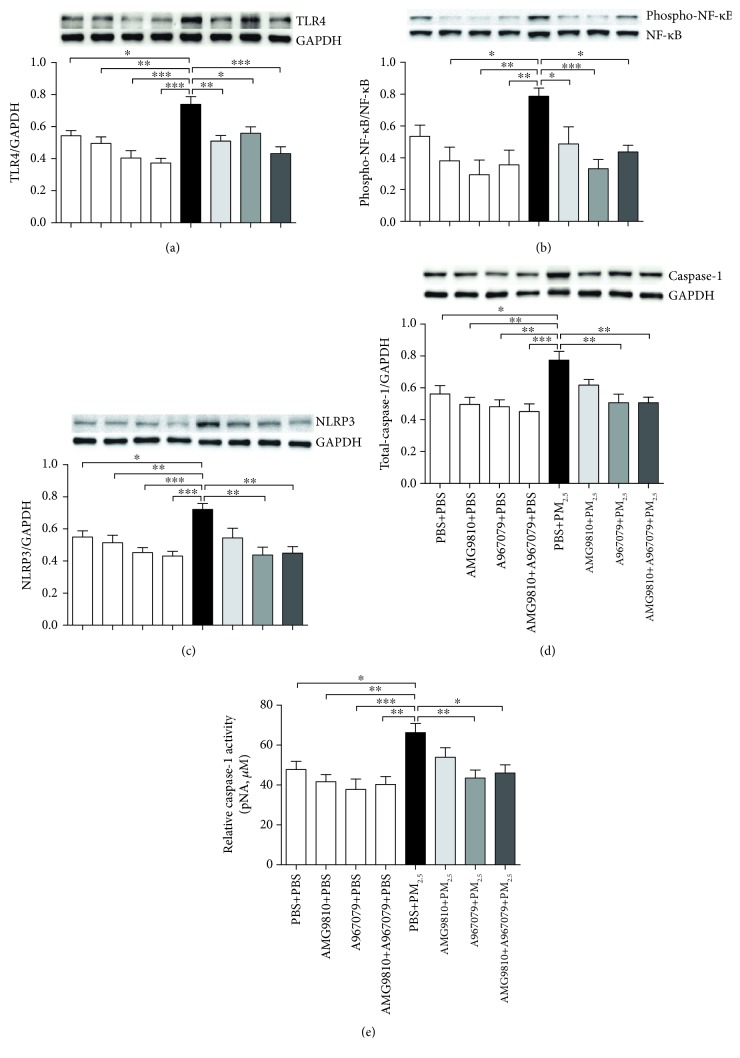
Western blot analysis of the relative protein expression of TLR4 (a), NLRP3 (c), and caspase-1 (d) to glyceraldehyde-3-phosphate dehydrogenase (GAPDH) and the ratio of phosphorylated (phospho) NF-*κ*B to total NF-*κ*B (b) in mouse lung tissue homogenates. Each panel shows a representative Western blot. Individual and relative activity of caspase-1 in lung tissue homogenates (e). ^∗^*P* < 0.05, ^∗∗^*P* < 0.01, and ^∗∗∗^*P* < 0.001 compared with PBS+PM_2.5_ mice.

There were increases in the protein level of TLR4 and the phosphorylation level of NF-*κ*B in PBS-pretreated PM_2.5_-instilled mice compared to PBS-pretreated PBS-instilled mice (Figures 8(a) and 8(b)). In PBS-instilled mice, pretreatment with AMG9810, A967079, or their combination had no effects on TLR4 protein and NF-*κ*B phosphorylation compared with that in PBS-pretreated PBS-instilled mice (Figures 8(a) and 8(b)). In PM_2.5_-instilled mice, pretreatment with AMG9810, A967079, or their combination inhibited the TLR4 protein and NF-*κ*B phosphorylation compared to that in PBS-pretreated PM_2.5_-instilled mice (Figures 8(a) and 8(b)).

PM_2.5_ instillation increased the protein of NLRP3 and caspase-1 and the activity of caspase-1 compared to PBS-instilled mice (Figures 8(c)–8(e)). There were no effects on NLRP3 and the caspase-1 protein and caspase-1 activity by pretreatment with AMG9810, A967079, or their combination in PBS-instilled mice. In PM_2.5_-instilled mice, A967079 alone and in combination with AMG9810 inhibited the NLRP3 protein, caspase-1 protein, and activity (Figures 8(c)–8(e)).

## 4. Discussion

In the present study, we demonstrated that PM_2.5_ intranasal instillation induced lung inflammation, AHR, and oxidative stress in mice and separate or combined pretreatment with AMG9810 or/and A967079 attenuated PM_2.5_-induced lung inflammation, AHR, and oxidative stress. All pretreatments mediated the expression of mitochondrial fission/fusion proteins and inhibited the activation of the TLR4/NF-*κ*B pathway, and A967079 alone, and combined with AMG9810 also inhibited the NLRP3/caspase-1 pathway. Thus, we conclude that inhibition of TRPA1 or combined inhibition of TRPA1 and TRPV1 showed better inhibitory effects on PM_2.5_-induced lung injury than that of TRPV1 through regulating the mitochondrial fission/fusion and inhibiting the TLR4/NF-*κ*B and NLRP3/caspase-1 pathways.

The TRPV1 and TRPA1 channels are coexpressed in mouse bronchopulmonary afferent neurons (jugular/nodose), as well as in nonneuronal cells including mouse AECs and ASM cells. The activation of TRPV1 and TRPA1 channels by noxious stimuli generally induced distinct neurogenic inflammation, which promoted the recruitment of immune cells and the activation of structural cells such as AECs and fibroblasts that reside within damaged tissues to release inflammatory factors [20]. Similarly, PM_2.5_ interacts with structural cells and immune cells in airways and induces infiltration of inflammatory cells and production of oxidative stress which can directly activate TRPA1 and TRPV1 channels [21, 22]. In the acute PM_2.5_ instillation model, there were increased total cells, including macrophages, lymphocytes, and neutrophils in the BAL fluid. AMG9810 reduced total cells, KC, and IL-6, while A967079 alone, or in combination with AMG9810 reduced total cells, TNF-*α*, KC, IL-1*β*, and IL-6 in the BAL fluid. Therefore, the TRPA1 channel may play a more important role than the TRPV1 channel in PM_2.5_-induced lung inflammation.

AHR is generally caused by enhanced ASM contraction and airway narrowing in response to various stimuli and considered as a hallmark of allergic asthma [23]. Recent studies have shown that PM_2.5_ exposure aggravated AHR and allergic airway responses [2, 24]. AHR may also be evoked by neutrophilia [25] and increases of inflammatory cytokines such as TNF-*α* and IL-1*β* [23]. The activation of TRPV1 and TRPA1 triggers the release of neuropeptides which initiates ASM contraction manifested as cough, dyspnea, and AHR [26]. The inhibition of TRPV1 and TRPA1 abrogates AHR and leucocyte infiltration in airways [27, 28]. Similarly, our results showed that the administration of TRPV1 or/and TRPA1 antagonist significantly inhibited PM_2.5_-induced lung inflammation and prevented PM_2.5_-induced AHR.

PM_2.5_-induced oxidative stress, characterized by the imbalance between oxidant and antioxidant molecules, could lead to disruption of redox homeostasis and sustained tissue damage [4, 29, 30]. In this connection, excessive ROS promote oxidation of lipids, nucleic acids, and proteins, which can then impair cellular integrity and mitochondrial function and, in turn, can cause the production of mtROS [31]. MDA, a typical biomarker of lipid peroxidation, binds covalently to proteins and DNAs to weaken antioxidant enzyme activities and decay cell function [32]. H_2_O_2_, a major form of ROS, could produce enormous hydroxyl radicals (^·^OH) through iron redox cycling and could further accelerate the oxidation of lipids and impairment of membrane function [33]. Our study showed that PM_2.5_ instillation induced increases in MDA, H_2_O_2_, and mtROS levels. TRPV1 and TRPA1 can be directly activated by oxidizing substances through cysteine modification [21, 34]. Particularly, the sensitivity of TRPA1 to ROS is greater than TRPV1. TRPA1 can be activated by H_2_O_2_-derived ^·^OH [35], and TRPA1 has been suggested to function as a major sensor of oxidative stress in airway sensory neurons [36]. A recent study showed that the TRPA1 inhibitor (HC-030031) alleviated oxidative stress with decreases in MDA levels and inflammatory responses [37]. In our study, AMG9810 decreased lung H_2_O_2_ and mtROS levels and A967079 alone, or combined with AMG9810 reduced the levels of MDA, H_2_O_2_, and mtROS in the lung. Thus, we conclude that the effect of inhibition of TRPA1 is greater in suppressing PM_2.5_-induced oxidative stress in the lung than that of TRPV1.

Mitochondria are the major source of intracellular ROS and are also the main target of elevated ROS which may disrupt mitochondrial morphology and function [38]. Mitochondrial dynamic homeostasis relies on the delicate balance of the fission and fusion [9, 39]. The imbalance of mitochondrial fission and fusion induced by ROS leads to the cleavage of OPA1, activation of DRP1, and increases in fragmented discrete mitochondria, which further lead to the generation of more mtROS and cell apoptosis [8, 38, 40]. Consistent with previous studies [10, 41, 42], our results showed that PM_2.5_ instillation disrupted the balance of fission proteins (MFF and DRP1) and fusion proteins (MFN2 and OPA1). The intervention of AMG9810 or A967079 can partly regulate the expression of mitochondrial fission proteins and fusion proteins, and combined intervention has more comprehensive effects than separate intervention as indicated by decreasing MFF and DRP1 and increasing MFN2 and OPA1 proteins. Therefore, we assume that the block of TRPV1 and TRPA1 channels contributes to repairing PM_2.5_-induced mitochondrial damage.

In the present study, we also investigated potential signaling pathways by the antagonists of TRPV1 and TRPA1. Our previous study demonstrated that TLR4 signaling is involved in airway inflammation and AHR induced by PM_2.5_ [6]. Activated TLR4 drives the phosphorylation of NF-*κ*B signaling and promotes the expression of cytokines such as TNF-*α*, IL-1*β*, and IL-6 [43]. The results of our present study showed that AMG9810, A967079, and their combination inhibited the activation of the TLR4/NF-*κ*B pathway. Simultaneously, activated TLR4 contributed to the activation of the other PRR, the NLRP3 inflammasome [44]. Moreover, mtROS and impaired mtDNAs released by damaged mitochondrial could directly induce NLRP3 inflammasome activation [5, 44]. Intracellular Ca^2+^ enrichment and mobilization may also promote NLRP3 inflammasome activation [45]. The activation of TRPV1 and TRPA1 channels could evoke abundant Ca^2+^ influx into the cytoplasm [11]. Once activated, caspase-1 processes pro-IL-1*β* and pro-IL-18 into their bioactive forms to initiate inflammatory responses [44, 45]. In our experiments, we found that A967079 alone, or in combination with AMG9810 inhibited NLRP3/caspase-1 expression and caspase-1 activity with reduced IL-1*β*. Therefore, we assume that the block of TRPA1 alone, or in combination with TRPV1 may inhibit inflammatory responses driven by the NLRP3/caspase-1 pathway.

In summary, the present study has demonstrated that the TRPA1 channel played a more important role in PM_2.5_-induced lung inflammation, AHR, and oxidative stress than TRPV1. The inhibition of TRPA1 or combined inhibition of TRPV1 and TRPA1 may be more effective in inhibiting PM_2.5_-induced lung injury than that of TRPV1.

## Figures and Tables

**Table 1:** Biochemical analyses of PM_2.5_. Total organic carbon (TOC) (%) represents the proportion of TOC in the qualitative PM_2.5_ samples.

Assay item	Value
TOC (%)	14.38
Endotoxin (EU/*μ*g)	0.68
PAHs (mg/g)	710.56
Negative ion (mg/g)	
F^−^	1.434
Cl^−^	33.33
SO_4_^2-^	54.194
NO_3_^−^	49.332
Br^−^	0.354
PO_4_^3-^	1.606
Positive ion (mg/g)	
Li^+^	0.6952
Na^+^	25.442
NH_4_^+^	20.612
K^+^	4.2252
Mg^2+^	1.694
Ca^2+^	13.1648

PAHs: polycyclic aromatic hydrocarbons.

## Data Availability

The data used to support the findings of this study are available from the corresponding author upon request.

## Abbreviations

AHR: Airway hyperresponsiveness
TRPV1: Transient receptor potential vanilloid 1
TRPA1: Transient receptor potential ankyrin 1
PM_2.5_: Fine particulate matter
PRRs: Pattern recognition receptors
TLRs: Toll-like receptors
NLRs: Nucleotide binding domain leucine-rich repeat-containing receptors
ATP: Adenosine triphosphate
TLR4: Toll-like receptor 4
NLRP3: NLR family pyrin domain containing 3
DRP1: Dynamin-related protein 1
MFF: Mitochondrial fission factor
OPA1: Optic atrophy 1
MFN2: Mitofusin 2
ROS: Reactive oxygen species
mtROS: Mitochondrial reactive oxygen species
AECs: Airway epithelial cells
ASM: Airway smooth muscle
H_2_O_2_: Hydrogen peroxide
DEP: Diesel exhaust particles
NKA: Neurokinin A
CGRP: Calcitonin gene-related peptide
PBS: Phosphate-buffered saline
ACh: Acetylcholine
BAL fluid: Bronchoalveolar lavage fluid
KC: Chemokine (C-X-C motif) ligand 1
MDA: Malondialdehyde
H_2_O_2_: Hydrogen peroxide
^·^OH: Hydroxyl radicals
TOC: Total organic carbon
PAHs: Polycyclic aromatic hydrocarbons
GAPDH: Glyceraldehyde-3-phosphate dehydrogenase.

## References

[1] Li R., Zhou R., Zhang J. (2018). Function of PM2.5 in the pathogenesis of lung cancer and chronic airway inflammatory diseases (Review). *Oncology Letters*.

[2] Ogino K., Nagaoka K., Okuda T. (2017). PM2.5-induced airway inflammation and hyperresponsiveness in NC/Nga mice. *Environmental Toxicology*.

[3] Zhao Y. X., Zhang H. R., Yang X. N. (2018). Fine particulate matter-induced exacerbation of allergic asthma via activation of T-cell immunoglobulin and mucin domain 1. *Chinese Medical Journal*.

[4] Ovrevik J., Refsnes M., Lag M., Holme J. A., Schwarze P. E. (2015). Activation of proinflammatory responses in cells of the airway mucosa by particulate matter: oxidant- and non-oxidant-mediated triggering mechanisms. *Biomolecules*.

[5] Sandhir R., Halder A., Sunkaria A. (2017). Mitochondria as a centrally positioned hub in the innate immune response. *Biochimica et Biophysica Acta (BBA) - Molecular Basis of Disease*.

[6] Xu M., Li F., Wang M. (2019). Protective effects of VGX-1027 in PM_2.5_-induced airway inflammation and bronchial hyperresponsiveness. *European Journal of Pharmacology*.

[7] Shadel G. S., Horvath T. L. (2015). Mitochondrial ROS signaling in organismal homeostasis. *Cell*.

[8] Angajala A., Lim S., Phillips J. B. (2018). Diverse roles of mitochondria in immune responses: novel insights into immuno-metabolism. *Frontiers in Immunology*.

[9] Gilkerson R. (2018). A disturbance in the force: cellular stress sensing by the mitochondrial network. *Antioxidants*.

[10] Guo Z., Hong Z., Dong W. (2017). PM_2.5_-induced oxidative stress and mitochondrial damage in the nasal mucosa of rats. *International Journal of Environmental Research and Public Health*.

[11] Dietrich A., Steinritz D., Gudermann T. (2017). Transient receptor potential (TRP) channels as molecular targets in lung toxicology and associated diseases. *Cell Calcium*.

[12] Gultekin F., Naziroglu M., Savas H. B., Cig B. (2018). Calorie restriction protects against apoptosis, mitochondrial oxidative stress and increased calcium signaling through inhibition of TRPV1 channel in the hippocampus and dorsal root ganglion of rats. *Metabolic Brain Disease*.

[13] Trevisan G., Benemei S., Materazzi S. (2016). TRPA1 mediates trigeminal neuropathic pain in mice downstream of monocytes/macrophages and oxidative stress. *Brain*.

[14] Yang H., Li S. (2016). Transient receptor potential ankyrin 1 (TRPA1) channel and neurogenic inflammation in pathogenesis of asthma. *Medical Science Monitor*.

[15] Bonvini S. J., Belvisi M. G. (2017). Cough and airway disease: the role of ion channels. *Pulmonary Pharmacology & Therapeutics*.

[16] Brozmanova M., Mazurova L., Ru F., Tatar M., Kollarik M. (2012). Comparison of TRPA1-versus TRPV1-mediated cough in guinea pigs. *European Journal of Pharmacology*.

[17] Alawi K. M., Aubdool A. A., Liang L. (2015). The sympathetic nervous system is controlled by transient receptor potential vanilloid 1 in the regulation of body temperature. *The FASEB Journal*.

[18] Huang Q., Chen Y., Gong N., Wang Y. X. (2016). Methylglyoxal mediates streptozotocin-induced diabetic neuropathic pain via activation of the peripheral TRPA1 and Nav1.8 channels. *Metabolism*.

[19] Li F., Wiegman C., Seiffert J. M. (2013). Effects of N-acetylcysteine in ozone-induced chronic obstructive pulmonary disease model. *PLoS One*.

[20] Bautista D. M., Pellegrino M., Tsunozaki M. (2013). TRPA1: a gatekeeper for inflammation. *Annual Review of Physiology*.

[21] Ogawa N., Kurokawa T., Mori Y. (2016). Sensing of redox status by TRP channels. *Cell Calcium*.

[22] Falcon-Rodriguez C. I., Osornio-Vargas A. R., Sada-Ovalle I., Segura-Medina P. (2016). Aeroparticles, composition, and lung diseases. *Frontiers in Immunology*.

[23] Sakai H., Suto W., Kai Y., Chiba Y. (2017). Mechanisms underlying the pathogenesis of hyper-contractility of bronchial smooth muscle in allergic asthma. *Journal of Smooth Muscle Research*.

[24] Wang X., Hui Y., Zhao L., Hao Y., Guo H., Ren F. (2017). Oral administration of *Lactobacillus paracasei* L9 attenuates PM_2.5_-induced enhancement of airway hyperresponsiveness and allergic airway response in murine model of asthma. *PLoS One*.

[25] McGovern T. K., Chen M., Allard B., Larsson K., Martin J. G., Adner M. (2016). Neutrophilic oxidative stress mediates organic dust-induced pulmonary inflammation and airway hyperresponsiveness. *American Journal of Physiology-Lung Cellular and Molecular Physiology*.

[26] Wallace H., Emir T. L. R. (2017). Airway pathogenesis is linked to TRP channels. *Neurobiology of TRP Channels*.

[27] Hox V., Vanoirbeek J. A., Alpizar Y. A. (2013). Crucial role of transient receptor potential ankyrin 1 and mast cells in induction of nonallergic airway hyperreactivity in mice. *American Journal of Respiratory and Critical Care Medicine*.

[28] Delescluse I., Mace H., Adcock J. J. (2012). Inhibition of airway hyper-responsiveness by TRPV1 antagonists (SB-705498 and PF-04065463) in the unanaesthetized, ovalbumin-sensitized guinea pig. *British Journal of Pharmacology*.

[29] Gawda A., Majka G., Nowak B., Marcinkiewicz J. (2017). Air pollution, oxidative stress, and exacerbation of autoimmune diseases. *Central European Journal of Immunology*.

[30] Sies H. (2015). Oxidative stress: a concept in redox biology and medicine. *Redox Biology*.

[31] Sinha K., Das J., Pal P. B., Sil P. C. (2013). Oxidative stress: the mitochondria-dependent and mitochondria-independent pathways of apoptosis. *Archives of Toxicology*.

[32] Barrera G., Pizzimenti S., Daga M. (2018). Lipid peroxidation-derived aldehydes, 4-hydroxynonenal and malondialdehyde in aging-related disorders. *Antioxidants*.

[33] Larosa V., Remacle C. (2018). Insights into the respiratory chain and oxidative stress. *Bioscience Reports*.

[34] Naziroglu M., Braidy N. (2017). Thermo-sensitive TRP channels: novel targets for treating chemotherapy-induced peripheral pain. *Frontiers in Physiology*.

[35] Andersson D. A., Gentry C., Moss S., Bevan S. (2008). Transient receptor potential A1 is a sensory receptor for multiple products of oxidative stress. *The Journal of Neuroscience*.

[36] Yamamoto S., Shimizu S. (2016). Significance of TRP channels in oxidative stress. *European Journal of Pharmacology*.

[37] Wang Z., Wang M., Liu J. (2018). Inhibition of TRPA1 attenuates doxorubicin-induced acute cardiotoxicity by suppressing oxidative stress, the inflammatory response, and endoplasmic reticulum stress. *Oxidative Medicine and Cellular Longevity*.

[38] Cid-Castro C., Hernandez-Espinosa D. R., Moran J. (2018). ROS as regulators of mitochondrial dynamics in neurons. *Cellular and Molecular Neurobiology*.

[39] Patrushev M. V., Mazunin I. O., Vinogradova E. N., Kamenski P. A. (2015). Mitochondrial fission and fusion. *Biochemistry*.

[40] Szabo A., Sumegi K., Fekete K. (2018). Activation of mitochondrial fusion provides a new treatment for mitochondria-related diseases. *Biochemical Pharmacology*.

[41] Li R., Kou X., Geng H. (2015). Effect of ambient PM_2.5_ on lung mitochondrial damage and fusion/fission gene expression in rats. *Chemical Research in Toxicology*.

[42] Zhang J., Liu J., Ren L. (2018). PM_2.5_ induces male reproductive toxicity via mitochondrial dysfunction, DNA damage and RIPK1 mediated apoptotic signaling pathway. *Science of The Total Environment*.

[43] Kuzmich N. N., Sivak K. V., Chubarev V. N., Porozov Y. B., Savateeva-Lyubimova T. N., Peri F. (2017). TLR4 signaling pathway modulators as potential therapeutics in inflammation and sepsis. *Vaccines*.

[44] Poudel B., Gurung P. (2018). An update on cell intrinsic negative regulators of the NLRP3 inflammasome. *Journal of Leukocyte Biology*.

[45] Prochnicki T., Mangan M. S., Latz E. (2016). Recent insights into the molecular mechanisms of the NLRP3 inflammasome activation. *F1000Research*.

